# Quality of Life of Celiac Patients in Brazil: Questionnaire Translation, Cultural Adaptation and Validation

**DOI:** 10.3390/nu10091167

**Published:** 2018-08-25

**Authors:** Claudia B. Pratesi, Winfred Häuser, Rosa Harumi Uenishi, Nicole Selleski, Eduardo Yoshio Nakano, Lenora Gandolfi, Riccardo Pratesi, Renata Puppin Zandonadi

**Affiliations:** 1 Interdisciplinary Laboratory of Biosciences and Celiac Disease Research Center, School of Medicine, University of Brasilia, 70910-900 Brasilia, DF, Brazil; claudiapratesi@hotmail.com (C.P.P.); rosa.uenishi@gmail.com (R.H.U.); selleskinicole@gmail.com (N.S.); lenoragandolfi1@gmail.com (L.G.); pratesiunb@gmail.com (R.P.); 2 Post-graduate Program in Health Sciences, School of Health Sciences, University of Brasilia, Brasilia 70910-900, DF, Brazil; 3 Department of Internal Medicine I, Klinikum Saarbrücken and Department of Psychosomatic Medicine and Psychotherapy, Technische Universität München, 80333 München, Germany; whaeuser@klinikum-saarbruecken.de; 4 Department of Statistics, University of Brasilia, 70910-900 Brasilia, DF, Brazil; eynakano@gmail.com; 5 Department of Nutrition, School of Health Sciences, University of Brasilia, 70910-900 Brasilia, DF, Brazil

**Keywords:** Brazilian CD-QoL, quality of life, celiac disease, questionnaire

## Abstract

(1) Background: Celiac disease (CD) is a common autoimmune disorder. The manifestations of the disease and the obligatory life-long gluten-free diet (GFD) are associated with the impairment of patients’ quality of life. Therefore, the present study aimed to translate, culturally adapt and validate a celiac disease quality of life (CD-QoL) questionnaire and apply it to a representative number of Brazilian CD patients. (2) Methods: A cross-cultural Brazilian-Portuguese version of the CD-QoL was developed according to revised international guidelines. The questionnaire was administered to 450 celiac patients. The reliability, reproducibility and validity were studied. (3) Results: The Brazilian CD-QoL questionnaire presents valid measures of reproducibility and internal consistency. Early diagnosis is related to higher scores of Brazilian CD-QoL social, sub- and total scale. There was a positive correlation between higher education level and higher QoL. Individuals with partners tend to have a better emotional subscale of QoL. CD-patients who follow a strict GFD have highest QoL scale values. Men scored higher than women on the CD-QoL. All results were statistically significant except for the gastrointestinal subscale. (4) Conclusions: Brazilian CD-QoL allows comparative research between different celiac populations in the world. QoL research will help in the development of effective strategies to improve Brazilian celiac patients’ quality of life.

## 1. Introduction

Celiac disease (CD) is an autoimmune enteropathy that occurs in genetically susceptible individuals through the consumption of gluten and affects approximately 1% of the population worldwide [1,2]. Although there are asymptomatic patients, the disorder is generally characterized by the combination of gluten-dependent symptoms such as diarrhea, malabsorption, abdominal pain and weight loss. Extra-intestinal manifestations may also occur, such as arthralgia, osteoporosis, chronic fatigue, iron-deficiency anemia, depression and in some cases infertility and miscarriage [3,4]. Despite significant advances in understanding the physiopathology and treatment of celiac disease, the only available treatment continues to be a strict lifelong gluten-free diet (GFD) [5]. The dietary restrictions and the symptoms can significantly impact the patient’s quality of life (QoL) [6,7,8,9]. 

The knowledge of the QoL is essential for the evaluation and implementation of measures that can reduce the physical, emotional and social burden on patients affected. In this context, the celiac disease quality of life (CD-QoL) questionnaire is a valuable tool to evaluate the difficulties faced by the celiac patient, regarding symptoms, GFD and social exclusion [6,7]. It is also essential to provide data to promote the improvement of public health policies and consequently, the wellbeing of CD patients.

Quality of life questionnaires specific for celiac patients have been validated and applied in Germany (original), Italy, Turkey and France [7,8,9,10]. However, none have been adapted, validated and performed to assess the QoL of Brazilian CD patients. 

It is important to highlight that the prospect for celiac patients in Brazil is not optimal and therefore their QoL is impacted [11]. Although there has been an increase in the number of gluten-free products in Brazil, the market is still much smaller than that of conventional products. Finding gluten-free products with adequate taste and nutritional value at an affordable price is a challenge for celiac patients. Additionally, eating out becomes an even bigger challenge for celiac patients, due to lack of information about ingredients used and the risk of cross-contamination [12,13].

Therefore, the present study aimed to translate, culturally adapt, validate and apply a CD-QoL questionnaire to a representative sample of the Brazilian celiac population. We expect that the present study will allow future comparative research between different celiac populations. Potentially, this could also help health professionals and governmental institutions develop effective strategies to improve the quality of life of Brazilian celiac patients.

## 2. Materials and Methods 

The study was developed in six steps: (i) translation, (ii) cultural adaptation, (iii) validation of the questionnaire, (iv) evaluation of the internal consistency and reproducibility of the QoL, (v) application of the questionnaire to Brazilian celiac patients and (vi) statistical analysis. The study was approved by the Health Sciences Ethics Committee, University of Brasilia, No. 69119317.3.0000.0030 and followed the guidelines established by the Declaration of Helsinki.

### 2.1. Questionnaire 

This study followed the original version of the CD-QoL by Häuser et al. [7], which consists of 28 items divided into four areas (with seven items in each): (i) emotions (depressed, restless, relaxed, happy, physically fatigued, tearful); (ii) gastrointestinal symptoms (loose bowels, sudden urge for bowel movement, abdominal cramps, bloating, incomplete bowel evacuation, belching, nausea); (iii) concerns (being diagnosed too late, fear of medical examinations, afraid of cancer, lack of medical expertise, problems with health providers, inheritance of the disease to children); (iv) social (lack of understanding by colleagues, difficulties in recreation/sports, professional limitations, lack of understanding by family/friends, invitation/dinner, feeling of exclusion from others, sexual activities). 

In the original questionnaire, responses were scored on a seven-point Likert scale in which “7” corresponds to the best function and “1” to the worst. The total CD-QoL score ranges from 49 to 196. The scores of each area range from 7 to 49, where a higher score indicates a better quality of life. All questions asked were based on the respondent’s experience in the past two weeks to reduce memory bias. We chose the questionnaire designed by Häuser et al. [7] due to its capacity to evaluate attitudes and perceptions of celiac patients, covering physical, social and emotional aspects. 

### 2.2. Translation, Cultural Adaptation and Validation

The translation and cultural adaptation of the instrument was done in three phases following an adaptation of the parameters established by the International Quality of Life Assessment (IQOLA) project methodology [14] and the Delphi method [15]. 

#### 2.2.1. Translation and Retranslation 

For the translation phase, two bilingual health professionals independently translated the CD-QoL questionnaire from English to Portuguese, emphasizing conceptual rather than literal translation. The English questionnaire was translated to a Brazilian Portuguese 7th-grade reading level to obtain a better understanding of the questions by the general population. After the first translation, both translators, along with two health professionals with extensive experience with CD, met to resolve any discrepancies and integrate both translations into a single version. A single version was retranslated from Brazilian Portuguese to English, by two different bilingual translators working independently from each other, to confirm its accuracy to the original questionnaire. Lastly, the four translators jointly checked the final questionnaire version for accuracy. An adapted and modified version of the Delphi method [15] was used for the validation process. 

#### 2.2.2. Cultural Adaptation, Semantic Evaluation and Validation (First Step)

The validation of an instrument consists of a methodological procedure to evaluate its quality, which is related to the capacity of the instrument to accurately measure what it is intended to measure [16]. Therefore, the validation of the questionnaire occurred in two different steps. In the first step, the cultural adaptation, semantic evaluation and content validation were analyzed by a panel composed of professionals and researchers recognized in their areas. The expert panel consensus helps to define the instrument items which should be maintained, revised, or excluded [17,18]. 

Twelve health professionals were contacted by email. They were invited to participate and assist with cultural adaptation and semantic evaluation of the questionnaire; ten agreed to participate. Following their consent, participants received an email with a link to the questionnaire in Brazilian Portuguese that was placed on SurveyMonkey^®^ (San Mateo, CA, USA), an online survey platform. The online survey contained all 28 questions translated to Portuguese. The judges rated the questions on a five-point Likert Scale for clarity and, when applicable, made suggestions to improve the questionnaire regarding cultural adaptation, comprehension and clarity.

The mean grade for the evaluation of clarity and content validation of each item and semantic evaluation was calculated considering the answers provided by the experts. The degree of agreement among the experts for the assessment of importance and clarity of the items was evaluated through the Kendall (W) coefficient of concordance, which ranges from 0 to 1. High W-values (W ≥ 0.66) indicate that the experts applied the same standards of evaluation as opposed to Low W-values, which suggest disagreement among the experts [16]. The criteria established for the approval of the item was a minimal of 80% of agreement between the experts (W-values ≥ 0.8) [12]. Items considered unclear were rewritten in a different manner and subject to further evaluation by the experts. Once the ten participants approved all questions, two bilingual translators met and compared the new Brazilian Portuguese version of the questionnaire to the original version in English. This phase ensured that the Brazilian Portuguese version of the questionnaire was of appropriate cultural relevance while maintaining its fidelity to the original version.

#### 2.2.3. Pilot Test (Second Step)

In the pilot study, the new version of CD-QoL was applied to four celiac subjects that had been patients at the University of Brasilia Hospital (HUB) out-patient celiac clinic for over ten years. These patients rated the questions for reliability, clarity and ease of comprehension. Subsequently, a second evaluation was carried out a week later with four other longstanding celiac patients from the clinic. When the reliability, clarity and ease and of comprehension of each question achieved the score (80% of agreement) the step of internal consistency and reproducibility of the QoL was achieved.

#### 2.2.4. Internal Consistency and Reproducibility of the Brazilian CD-QoL

The internal consistency and reproducibility of the Brazilian CD-QoL were evaluated using 18 celiac patients’ (who did not participate in previous phases) responses. The celiac patients answered the Brazilian CD-QoL and after one week they were invited to answer the same questionnaire again. The Cronbach alpha measure evaluated the internal consistency of the QoL subscales. The test-retest reliability (reproducibility) of the questionnaire was verified using the Pearson correlation coefficient and the intra-class correlation coefficient (ICC).

### 2.3. Brazilian CD-QoL Application

The final step was to place the Brazilian Portuguese CD-QoL questionnaire on the SurveyMonkey^®^ platform and apply it to a representative number of Brazilian celiac patients. The first page of the survey contained the consent form that included the established exclusion/inclusion criteria; where participants had to be 18 years of age or older and have been positively diagnosed by a physician for over a year. At that point, participants gave their consent to participate. Those that did not agree to participate were directed to a page thanking them for their time; while those that agreed, were directed to the first page of the survey containing ten social demographic questions. The third part of the survey consisted of applying the 28 translated and culturally adapted questions to Brazilian celiac patients.

### 2.4. Psychometric Evaluation, Validation and Statistics 

The statistical analysis was carried out following the score proposed by the original study by Häuser et al. research [7] where a higher score indicates a higher quality of life. Questions left blank where substituted by a median value of the corresponding dimensions. The total score was calculated for each demographic and clinical dimension. If more than 25% of the questions were left blank the questionnaire was eliminated from the analysis.

The descriptive statistics (mean, median, standard deviation, floor effect and ceiling effect) of the subscales of the Celiac Disease Questionnaire (CDQ) were presented. Student’s *t*-test and Variance Analysis (ANOVA) followed by Tukey post-hoc analysis was used to compare the values of the CDQ subscales with the variables of interest. All tests considered two-tailed hypotheses with a significance level of 5%. Confirmatory factor analysis was used to assess the factor validity. The Root Mean Square Error of Approximation (RMSEA) and the Chi-squared test of minimum discrepancy [19] evaluated the factor validity. The RMSEA ranges from 0 to 1, with smaller values indicating better model fit. A value of 0.06 or less is indicative of an acceptable model fit [20]. The statistical analyses were performed using IBM SPSS (Statistical Package for Social Sciences) version 22 (IBM SPSS Statistics for Windows, Version 22.0. IBM Corp, Armonk, NY, USA) and IBM SPSS AMOS (Analysis of Moment Structures) version 22 ( Amos (Version 22.0), IBM SPSS, Chicago, IL, USA).

## 3. Results

### 3.1. Translation, Cultural Adaptation, Semantic Evaluation and Content Validation 

**Figure 1:**
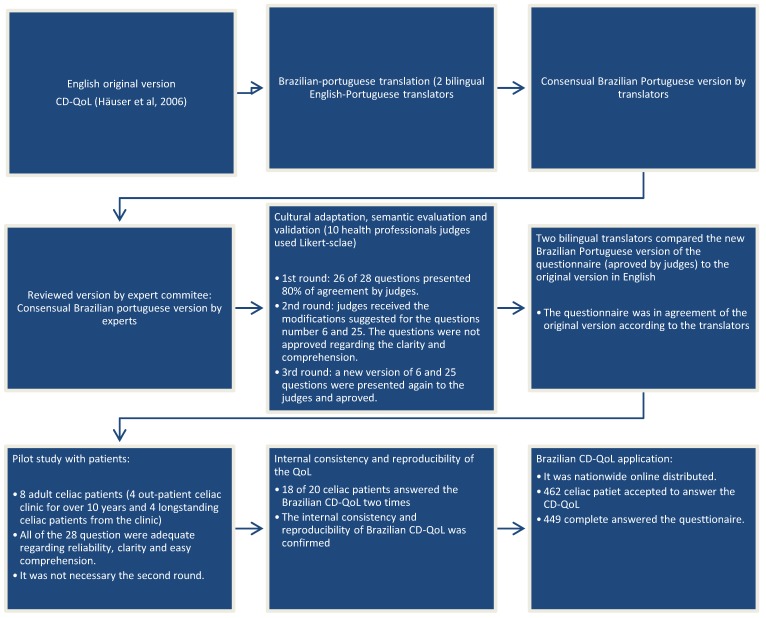
Process stages of the Brazilian celiac disease questionnaire of quality life (CD-QoL).

The summary of stages of the Brazilian questionnaire process is displayed in Figure 1. The Brazilian CD-QoL (Supplementary Material S1) was constructed considering the translation/retranslation and suggestions made by the experts and celiac patients in the pilot test. After the translation/retranslation steps, the first stage of the content validation and the semantic evaluation was performed by judges and they decided to maintain all of the 28 questions with cultural and semantic adaptations since we opted to follow an existing CD-QoL questionnaire [7]. In total, three rounds of evaluations were necessary to obtain agreement among the experts for the content validation and semantic evaluation. After that, a pilot study with eight celiac patients was conducted. In the first round of the questionnaire, questions were considered adequate regarding reliability, clarity and easy comprehension.

#### Internal Consistency, Construct Validity and Reproducibility of the Brazilian CD-QoL

The concordances of the answers (internal consistency) were verified by the Cronbach’s alpha measure (Table 1). All four domains of the CD-QoL indicated good internal consistency (Cronbach’s alpha > 0.7). 

The test-retest reliability (reproducibility) of the questionnaire was verified using the paired *t* test, Pearson correlation coefficient and the intraclass correlation coefficient (Table 2). None of the four domains composing the final questionnaire showed significant divergence among the evaluators (*p* > 0.05 in the paired *t* test). The Pearson coefficient and Interclass correlation coefficient (ICC) are ideally more substantial and significant (*p* < 0.05). Therefore, the questionnaire presents proper measures of reproducibility.

### 3.2. CD-QoL Application

During the period from July to October of 2017, a link to the Brazilian CD-QoL was distributed nationwide by email to multiple Brazilian Celiac Associations (*Associação dos Celíacos do Brasil—Acelbra; and Federação Nacional das Associações de Celíacos do Brasil Fenacelbra*). Those associations either emailed the link to CD patients registered with them or published the link to the survey through social media that was subsequently shared by members. In addition to Brazilian Celiac Associations, dietitians and gastroenterologists were also asked to distribute the link to their CD patients. Therefore, we used a convenience sample to perform the present study.

A total of 462 participants from 18 out of 26 Brazilian States agreed to answer the questionnaire. The eight Brazilian States not represented were those with no Celiac Associations. Of the 462 questionnaires, 12 were excluded because they were not filled out. The remaining 450 questionnaires responses were analyzed. The questionnaire took an average of six minutes to be completed. Characteristics of the responders and their association with the CD-QoL subcategories are presented in Table 3. 

We divided the marital status category into either *in a stable relationship* (married or with a live-in partner) or *not in a stable relationship* (those that are single, divorced, or widowed). We also divided the Gluten-Free Diet category into two. Only participants that answered “always” were considered to be on a GFD, those that responded, “almost always”, “almost never”; “never” and “sometimes”, were considered not to be on a GFD.

Participants over the age of 40 presented higher values on the scale (statistically significant only for the total scale and the social subscale). Early diagnosis is related to higher scores on the social, sub- and total scale. There was a positive correlation between higher education level and higher QoL.

Individuals with partners tend to have a better QoL emotional subscale score (represent higher values on the scale). Regarding other subscales, the marital aspect did not influence the QoL. Individuals that follow a strict GFD have higher QoL scale values (except for the worries subscale) and those who do not take antidepressants have a higher quality of life.

The men’s scores for the CD-QoL were higher than the women’s. All results were statistically significant except for the gastrointestinal subscale, where there is no significant difference between women and men (*p* > 0.05). 

## 4. Discussion

Health is defined as “a state of complete physical, mental and social well-being” [21]. According to the World Health Organization, to achieve optimal health, it is essential to comprehend the patient’s perception of quality of life [22]. The Celiac Disease-specific Quality of Life Scale is an important and cost-effective tool to understand aspects related to the QoL of celiac patients. The scale helps us understand elements present in daily choices, management of mental and physical well-being as well as the social limitations imposed by this chronic disease due to the necessity for lifelong commitment [7,9]. 

When the quality of life assessment instrument is used in a variety of cultural settings, it is imperative to establish whether the same aspects of life are equally important to the group studied. Groups of people, particularly in different cultures, are likely to assign different levels of importance to various aspects of their life; therefore, cultural and semantic validation is crucial [23]. To the best of our knowledge, our study is the first characterization of the emotional, worries, gastrointestinal and social features related to the quality of life in CD Brazilian adults. In this context, we validated the first specific QoL instrument for CD patients in Brazilian Portuguese, based on the Hauser et al. [7] instrument.

The linguistic validation process (translation and retranslation) is recommended when the original instrument is developed in a language other than the target language and there is no translated and validated version in the target language [22]. Therefore, the first step of this study was to translate/retranslate the original version of CD-QoL to English/Portuguese/English. The translation was followed by the cross-cultural adaptation process that followed the guidelines predominant in the literature [24,25]. To acquire a reliable instrument, it is also vital to perform a semantic evaluation, which measures the comprehension of the instrument. This step ensures the instrument is clear and easy to understand [17]. The Brazilian Portuguese versions of the instrument demonstrate cultural and semantic adequacy and therefore represent the first Brazilian Portuguese version developed. A pilot study was conducted to evaluate the reliability and internal consistency of the instrument with celiac patients. 

Reliability is an estimate of the instrument’s ability to reproduce results provided that no change in the outcome has taken place [22]. In this study, it was measured with internal consistency and test-retest stability. The internal instrument consistency is an estimate of the extent to which the included items of a score correlate with each other. Cronbach’s alpha coefficient was used for this estimate for all patients, in all domains of the CD-QoL at baseline [26]. Internal consistency was considered acceptable when Cronbach’s alpha was 0.70 or higher [27]. None of the 28 items composing the final questionnaire showed significant divergence among the evaluators (>0.05 in the Cronbach alpha test) (Table 1). The 28-items of the CD-QoL show reliable internal consistency; therefore, the questionnaire presents good measures of reproducibility, which indicates that similar results under consistent conditions are reproducible. 

Once validated, the instrument was sent nationwide to Brazilian celiac patients to evaluate their quality of life. Similar to the other studies of CD-QoL in different countries [7,8,9], most of the respondents in this study were female (95%). It was expected, since the CD prevalence is higher in female patients than male patients [1,3,28] and women tend to be more concerned about health and participate more often in health studies [29,30,31,32]. The study conducted by Ramirez-Cervantes et al. [33] also had more responses from female participants (75%) compared to male participants in Mexico. In Brazil, Castilhos et al. [32] conducted a study with celiac patients that were registered in the Southern Brazilian Celiac Association (ACELPAR), which also showed a low rate of male participation (6.8%). They attribute these findings to the low participation rate of men in the ACELPAR meetings where the questionnaires were administered and more broadly, to the low interest in their health when compared to women [32].

In the present study, women’s scores for the CD-QoL were lower than men’s scores, except for the gastrointestinal subscale. Studies have shown that CD women experience a lower level of quality of life than men [13,34,35,36]. Women also report more distress caused by daily life restrictions and perceive a higher burden with CD than men [35].

Individuals that follow a strict GFD have higher QoL scale values ​​ (except for subscale worries). The strict adherence to the GFD tends to enhance physical and physiological aspects. Despite the food restriction, social wellbeing aspects can also be achieved, since symptoms and other conditions related to CD tend to be improved with the treatment adherence [37]. In line with previous studies, we found that diet-compliant CD patients (with an internal LoC (locus of control)) had a better QoL than noncompliant patients [7,9,32,38,39,40]. According to Wagner et al. [38], the GFD compliance is essential for celiac patients to obtain optimal QoL. Psychological and educational support is also essential for patients that are having difficulty adhering to a GFD [32,38]. According to Castilhos et al. [32], most of the CD patients felt well informed, showed no declining trend and showed no constant worry about their food. They believed that these results are related to the information and support received by the celiac association (ACELPAR). These results reinforce the importance of information and support to improve the QoL of CD patients.

Other studies showed similar results to ours; Wagner et al. (Wagner et al., 2008 [38]) showed that better physical health, lower CD-associated burden and fewer social problems, were found in participants who had a longer period since being diagnosed, indicating the importance of the earliest possible diagnosis. Ramírez-Cervantes et al. [33] also concluded that, at the time of diagnosis, CD patients had a reduced quality of life, compared to those with a longer diagnosis period—likely due to better knowledge about the disease and acceptance of the lifestyle. In the study conducted by Castilhos et al. [32], patients newly diagnosed were compared with those who had the longer diagnosis. The study showed that patients that were longer diagnosed had better QoL, suggesting that the limitations imposed by disease and GFD influence the patients QoL. They also indicate that over the years, there is a better adaptation to the restrictions imposed by the treatment [32]. Another study of Brazilian CD patients showed that the longer the time since diagnosis, the lower the chances that these patients had positive serological tests, indicating a better comprehension and adherence to the treatment [11]. 

Several studies highlight the positive role families can play in providing support to patients when adopting a GFD, as well as the support in coping with CD [13,41,42]. This active support provided is likely the reason why those “with partner” under marital status scored higher in the emotional subscale CD-QoL.

Past research has demonstrated that a higher educational level significantly contributes to the patient’s physical and social function, health perception and mental health. Low education amplifies the adverse effects of many chronic medical conditions due to lack of knowledge [43,44,45,46]. Our results, which corroborate the above findings, showed a positive correlation between higher education level and higher QoL. The education level tends to be associated with higher socioeconomic status [44]. A study suggests that income modulates both health-seeking behavior and access to health care [47], which is related to higher QoL.

A potential limitation to our study was selection bias created by the manner in which the survey was disseminated, that is, over the internet and with the use of email and social media. According to a census by the Brazilian Institute of Geography and Statistics (IBGE), approximately half of the Brazilian population 25 years of age and younger have less than eighth-grade education [48]. However, 56% of our respondents had a college degree or above, which is not representative of the Brazilian population. Consequently, there was also a selection bias regarding the socioeconomic level of the respondents, which is much higher than the national average. 

## 5. Conclusions

There is growing interest in assessing the outcome of CD, given the chronic nature of the disease. The development of a self-administered Brazilian-Portuguese CD-QoL instrument that captures the perceptions and concerns of CD individuals is an important step forward in the care of these patients. The Brazilian-Portuguese CD-QoL questionnaire version presents good measures of reproducibility and internal consistency. In Brazil, the time since diagnosis, higher education level, strict adherence to GFD and male gender are related to the highest scores of CD-QoL. Knowledge of the quality of life is important to help implement effective strategies to improve Brazilian celiac patients’ quality of life and for reducing the physical, emotional and social burden on them. Besides the Brazilian CD-QoL, an Italian, German, French and Turkish version of the CD-QoL have been published, which allows for comparative research between different celiac populations in the world. 

## Figures and Tables

**Table 1:** Descriptive and internal consistency of the Brazilian CD-QoL.

	Mean (SD)	Median (IQR)	Range	Floor Effect (%)	Ceiling Effect (%)	Internal Consistency (Cronbach’s Alpha)
Emotion	26.70 (9.55)	27 (20–34)	7–49	1.3%	0.2%	0.927
Social	31.15 (8.18)	32 (25–38)	10–48	0%	0%	0.703
Worries	27.25 (9.85)	27 (20–34)	7–49	0.4%	0.9%	0.832
Gastrointestinal	34.87 (8.61)	36 (29–41)	10–49	0%	2.4%	0.793
Total Score	119.79 (30.16)	120 (99–142)	51–181	0%	0%	0.925

SD—Standard deviation; IQR—Interquartile range. The factor/construct validity was examined by confirmatory factor analysis. The four domains (emotion, social, worries and gastrointestinal) presented a good fit in the confirmatory factor analysis (Root Mean Square Error of Approximation (RMSEA) = 0.016 and *χ*^2^ = 309.04, d*f* = 278, *p* = 0.097).

**Table 2:** CD-QoL scale reproducibility (*n* = 18 participants).

	Score			Pearson Correlation		Intraclass Correlation Coefficient	
	Phase 1 Mean (SD)	Phase 2 Mean (SD)	*p* *	Correlation	*p*	ICC	*p*
Emotion	25.50 (10.16)	27.06 (10.08)	0.153	0.905	<0.001	0.947	<0.001
Social	33.00 (9.17)	34.67 (7.08)	0.224	0.769	<0.001	0.850	<0.001
Worries	30.06 (7.68)	31.17 (8.54)	0.291	0.863	<0.001	0.923	<0.001
Gastrointestinal	37.06 (6.91)	35.17 (6.23)	0.247	0.486	0.041	0.647	0.018
Total	125.61 (27.11)	128.06 (27.08)	0.531	0.821	<0.001	0.905	<0.001

* paired *t*-test. ICC: Interclass correlation coefficient. *p* < 0.05 is statistically significant.

**Table 3:** Sub-scores of the CD-QoL scale subcategorized by sex, age, time of diagnosis, schooling, marital status and diet.

	Emotion	Social	Worries	Gastrointestinal	Total
	Mean (SD)	Mean (SD)	Mean (SD)	Mean (SD)	Mean (SD)
Gender *					
Women (*n* = 425)	26.42 (9.51)	30.08 (8.14)	26.80 (9.69)	34.75 (8.63)	118.55 (29.98)
Men (*n* = 25)	30.88 (9.03)	36.40 (6.57)	34.71 (9.96)	36.84 (8.33)	139.33 (25.95)
*p*	0.023	0.000	0.000	0.238	0.001
Age *					
39 and under (*n* = 271)	26.01 (9.13)	30.28 (7.93)	26.87 (9.64)	34.28 (8.45)	117.34 (28.98)
40 and over (*n* = 179)	27.74 (10.08)	32.48 (8.40)	27.82 (10.17)	35.77 (8.79)	123.52 (31.59)
*p*	0.065	0.006	0.032	0.074	0.036
Time of diagnosis *					
29 and under (*n* = 170)	25.27 (8.76)	29.97 (8.14)	26.75 (9.61)	33.99 (8.51)	115.72 (29.16)
30 and over (*n* = 273)	27.48 (9.92)	31.80 (8.20)	27.27 (9.93)	35.34 (8.67)	121.75 (30.58)
*p*	0.015	0.024	0.588	0.109	0.043
Educational level **					
Elementary (*n* = 26)	23.00 (10.92) ^a^	28.44 (6.68) ^a^	24.23 (10.78) ^a^	29.42 (8.68) ^a^	106.12 (30.32) ^a^
High School (*n* = 116)	24.10 (9.60) ^a,b^	29.29 (8.20) ^a,b^	25.69 (9.69) ^a,b^	32.69 (9.36) ^a,b^	111.57 (30.21) ^a,b^
College (*n* = 152)	27.32 (9.13) ^b,c^	31.34 (8.06) ^a,b^	27.29 (9.54) ^a,b^	35.43 (8.22) ^b,c^	121.01 (29.36) ^b,c^
Graduate & Post-grad (*n* = 156)	28.65 (9.14) ^c^	32.79 (8.16) ^b^	28.88 (9.90) ^b^	36.86 (7.67) ^c^	127.01 (28.86) ^c^
*p*	0.000	0.002	0.022	0.000	0.000
Marital status *					
With partners (*n* = 275)	27.51 (9.68)	31.69 (8.25)	27.30 (9.68)	35.36 (8.39)	121.78 (29.85)
Without partners (*n* = 175)	25.43 (9.22)	30.31 (8.02)	27.18 (10.14)	34.11 (8.91)	116.68 (30.47)
*p*	0.025	0.085	0.908	0.136	0.084
Gluten-free diet compliance *					
No (*n* = 51)	20.61 (9.37)	28.10 (7.43)	25.16 (10.97)	29.73 (8.71)	104.20 (30.00)
Yes (*n* = 399)	27.48 (9.30)	31.53 (8.20)	27.52 (9.68)	35.53 (8.38)	121.76 (29.64)
*p*	0.000	0.005	0.108	0.838	0.000
Antidepressant medicines *					
No (*n* = 371)	27.67 (9.37)	31.67 (7.98)	27.76 (9.78)	35.50 (8.49)	122.45 (29.63)
Yes (*n* = 79)	22.15 (9.09)	28.79 (8.72)	24.85 (9.90)	31.95 (8.63)	107.73 (29.81)
*p*	0.000	0.004	0.017	0.001	0.000

* Student *t*-test. ** Anova with Tukey post-hoc test. Groups with the same letters do not differ significantly. Different letters on the same column represent statistical differences.

## Supplementary Materials

The following are available online at http://www.mdpi.com/2072-6643/10/9/1167/s1. Supplementary Material S1: Brazilian CD-QoL in Portuguese.

## References

[1] Shamir R., Heyman M.B., Koning F., Wijimenga C., Gutierrez-Achury J., Catassi C., Gatti S., Fasano A., Discepolo V., Korponay-Szabó I.R. (2014). Celiac Disease. J. Pediatr. Gastroenterol. Nutr..

[2] Schuppan D., Junker Y., Barisani D. (2009). Celiac Disease: From Pathogenesis to Novel Therapies. Gastroenterology.

[3] Barada K., Abu Daya H., Rostami K., Catassi C. (2012). Celiac Disease in the Developing World. Gastrointest. Endosc. Clin. N. Am..

[4] Barker J.M., Liu E. (2008). Celiac disease: Pathophysiology, clinical manifestations and associated autoimmune conditions. Adv. Pediatr..

[5] Kaukinen K., Lindfors K., Mäki M. (2014). Advances in the treatment of coeliac disease: An immunopathogenic perspective. Nat. Rev. Gastroenterol. Hepatol..

[6] Zingone F., Iavarone A., Tortora R., Imperatore N., Pellegrini L., Russo T., Dorn S.D., Ciacci C. (2013). The Italian translation of the Celiac Disease-specific Quality of Life Scale in celiac patients on gluten free diet. Dig. Liver Dis..

[7] Häuser W., Gold J., Stein J., Caspary W.F., Stallmach A. (2006). Health-related quality of life in adult coeliac disease in Germany: Results of a national survey. Eur. J. Gastroenterol. Hepatol..

[8] Marchese A., Klersy C., Biagi F., Balduzzi D., Bianchi P.I., Trotta L., Vattiato C., Zilli A., Rademacher J., Andrealli A. (2013). Quality of life in coeliac patients: Italian validation of a coeliac questionnaire. Eur. J. Intern. Med..

[9] Aksan A., Mercanlıgil S.M., Häuser W., Karaismailoğlu E. (2015). Validation of the Turkish version of the Celiac Disease Questionnaire (CDQ). Health Qual. Life Outcomes.

[10] Pouchot J., Despujol C., Malamut G., Ecosse E., Coste J., Cellier C. (2014). Validation of a French version of the quality of life “Celiac Disease Questionnaire”. PLoS ONE.

[11] Machado J., Gandolfi L., Coutinho De Almeida F., Malta Almeida L., Puppin Zandonadi R., Pratesi R. (2013). Gluten-free dietary compliance in Brazilian celiac patients: Questionnaire versus serological test. Nutr. Clin. Diet. Hosp..

[12] Farage P., Puppin Zandonadi R., Cortez Ginani V., Gandolfi L., Pratesi R., de Medeiros Nóbrega Y.K. (2017). Content Validation and Semantic Evaluation of a Check-List Elaborated for the Prevention of Gluten Cross-Contamination in Food Services. Nutrients.

[13] Sverker A., Hensing G., Hallert C. (2005). ‘Controlled by food’—Lived experiences of coeliac disease. J. Hum. Nutr. Diet..

[14] Bullinger M., Alonso J., Apolone G., Leplège A., Sullivan M., Wood-Dauphinee S., Gandek B., Wagner A., Aaronson N., Bech P. (1998). Translating health status questionnaires and evaluating their quality: The IQOLA Project approach. International Quality of Life Assessment. J. Clin. Epidemiol..

[15] Okoli C., Pawlowski S.D. (2004). The Delphi method as a research tool: An example, design considerations and applications. Inf. Manag..

[16] De Lima T.C., Gallani M.C.B.J., de Freitas M.I.P. (2012). Content validation of an instrument to characterize people over 50 years of age living with human immunodeficiency virus/acquired immunodeficiency syndrome. Acta Paul. Enferm..

[17] Conti M.A., Scagliusi F., Kawamura De Oliveira Queiroz G., Hearst N., Cordás T.A. (2010). Cross-cultural adaptation: Translation and Portuguese language content validation of the Tripartite Influence Scale for body dissatisfaction. Cad. Saude Publ..

[18] Polit D.F., Beck C.T. (2004). Nursing Research: Principles and Methods.

[19] Kline R.B. (2011). Principles and Practice of Structural Equation Modeling.

[20] Hu L., Bentler P. (1999). Cutoff criteria for fit indices in covariance structure analysis: Conventional criteria versus new alternatives. Struct. Equ. Model..

[21] World Health Organization (2008). The Third Ten Years of the World Health Organization.

[22] Fagerdahl A.-M., Boström L., Ulfvarson J., Bergström G., Ottosson C. (2014). Translation and validation of the wound-specific quality of life instrument Cardiff Wound Impact Schedule in a Swedish population. Scand. J. Caring Sci..

[23] Saxena S., Carlson D., Billington R., Orley J. (2001). The WHO quality of life assessment instrument (WHOQOL-Bref): The importance of its items for cross-cultural research. Qual. Life Res..

[24] Guillemin F., Bombardier C., Beaton D. (1993). Cross-cultural adaptation of health-related quality of life measures: Literature review and proposed guidelines. J. Clin. Epidemiol..

[25] Beaton D.E., Bombardier C., Guillemin F., Ferraz M.B. (2000). Guidelines for the process of cross-cultural adaptation of self-report measures. Spine.

[26] Streiner D.L. (2003). Starting at the Beginning: An Introduction to Coefficient Alpha and Internal Consistency Starting at the Beginning: An Introduction to Coefficient Alpha and Internal Consistency. J. Pers. Assess..

[27] Streiner D.L., Norman G.R. (2008). Health Measurement Scales: A Practical Guide to Their Development and Use.

[28] Mäki M., Collin P. (1997). Coeliac disease. Lancet.

[29] Davidson D.J., Freudenburg W.R. (1996). Gender and Environmental Risk Concerns. Environ. Behav..

[30] Chen M. (2013). Consumers’ health and taste attitude in Taiwan. Br. Food J..

[31] Lee A.R., Wolf R., Contento I., Verdeli H., Green P.H.R. (2016). Coeliac disease: The association between quality of life and social support network participation. J. Hum. Nutr. Diet..

[32] Castilhos A.C., Gonçalves B.C., Macedo e Silva M., Lanzoni L.A., Metzger L.R., Kotze L.M.S., Nisihara R.M. (2015). Quality of life evaluation in celiac patients from Southern Brazil. Arq. Gastroenterol..

[33] Ramírez-Cervantes K.L., Remes-Troche J.M., del Pilar Milke-García M., Romero V., Uscanga L.F. (2015). Characteristics and factors related to quality of life in Mexican Mestizo patients with celiac disease. BMC Gastroenterol..

[34] Hallert C., Grännö C., Hultén S., Midhagen G., Ström M., Svensson H., Valdimarsson T. (2002). Living with coeliac disease: Controlled study of the burden of illness. Scand. J. Gastroenterol..

[35] Hallert C., Sandlund O., Broqvist M. (2003). Perceptions of health-related quality of life of men and women living with coeliac disease. Scand. J. Caring Sci..

[36] Zarkadas M., Cranney A., Case S., Molloy M., Switzer C., Graham I.D., Butzner J.D., Rashid M., Warren R.E., Burrows V. (2006). The impact of a gluten-free diet on adults with coeliac disease: Results of a national survey. J. Hum. Nutr. Diet..

[37] Bellini A., Zanchi C., Martelossi S., Di Leo G., Not T., Ventura A. (2011). Compliance with the Gluten-Free Diet: The Role of Locus of Control in Celiac Disease. J. Pediatr..

[38] Wagner G., Berger G., Sinnreich U., Grylli V., Schober E., Huber W.-D., Karwautz A. (2008). Quality of Life in Adolescents with Treated Coeliac Disease: Influence of Compliance and Age at Diagnosis. J. Pediatr. Gastroenterol. Nutr..

[39] Roma E., Roubani A., Kolia E., Panayiotou J., Zellos A., Syriopoulou V.P. (2010). Dietary compliance and life style of children with coeliac disease. J. Hum. Nutr. Diet..

[40] Häuser W., Stallmach A., Caspary W.F., Stein J. (2007). Predictors of reduced health-related quality of life in adults with coeliac disease. Aliment. Pharmacol. Ther..

[41] Jacobsson L.R., Friedrichsen M., Göransson A., Hallert C. (2012). Impact of an Active Patient Education Program on Gastrointestinal Symptoms in Women With Celiac Disease Following a Gluten-Free Diet. Gastroenterol. Nurs..

[42] Taylor E., Dickson-Swift V., Anderson K. (2013). Coeliac disease: The path to diagnosis and the reality of living with the disease. J. Hum. Nutr. Diet..

[43] Gazmararian J.A., Williams M.V., Peel J., Baker D.W. (2003). Health literacy and knowledge of chronic disease. Patient Educ. Couns..

[44] Everson S.A., Maty S.C., Lynch J.W., Kaplan G.A. (2002). Epidemiologic evidence for the relation between socioeconomic status and depression, obesity and diabetes. J. Psychosom. Res..

[45] Rojas-García A., Ruiz-Perez I., Rodríguez-Barranco M., Gonçalves Bradley D.C., Pastor-Moreno G., Ricci-Cabello I. (2015). Healthcare interventions for depression in low socioeconomic status populations: A systematic review and meta-analysis. Clin. Psychol. Rev..

[46] Elovainio M., Pulkki-Råback L., Jokela M., Kivimäki M., Hintsanen M., Hintsa T., Viikari J., Raitakari O.T., Keltikangas-Järvinen L. (2012). Socioeconomic status and the development of depressive symptoms from childhood to adulthood: A longitudinal analysis across 27 years of follow-up in the Young Finns study. Soc. Sci. Med..

[47] Mehra S., Leffler D.A., Pallav K., Tariq S., Shah S., Green P.H., Hansen J., Dennis M., Kelly C.P. (2011). Socioeconomic Status Influences Celiac Disease Diagnosis. Gastroenterology.

[48] IBGE (2010). Pesquisas de Orçamentos Familiares.

