# Supplementary material for: Quality of Life of Celiac Patients in Brazil: Questionnaire Translation, Cultural Adaptation and Validation
Source: Nutrients. 2018 Aug 25;10(9):1167. doi: 10.3390/nu10091167 (PMC6164351; doi:10.3390/nu10091167)
Supplement: Supplementary file 1 [file nutrients-10-01167-s001.pdf]

Brazilian CD-QoL in Portuguese

Questionário de doença celíaca (QDC)—índice relacionado a qualidade de viva e saúde de adultos portadores de doença celíaca.

Caracterização dos participantes

1. Sexo

F ( ) M( )

2. Idade

( ) 18–29
( ) 30–39
( ) 40–49
( ) 50–59
( ) >60

3. Idade quando diagnosticado

( ) < 5
( ) 5-10
( ) 11-20
( ) 21-30
( ) 31-40
( ) 41-50
( ) >50

4. Nível educacional

( ) Não estudou
( ) Da 1ª a 4ª série do ensino fundamental (antigo primário)
( ) Da 5ª a 8ª série do ensino fundamental (antigo ginásio)
( ) Ensino médio (2° grau) incompleto
( ) Ensino médio (2° grau) completo
( ) Ensino superior incompleto
( ) Ensino superior completo
( ) Pós-Graduação

5. Estado civil

( ) Solteiro(a)
( ) Casado(a) / união estável
( ) Divorciado (a)
( ) Viúvo(a)

6. Ocupação

( ) Estágio
( ) Emprego fixo particular
( ) Emprego autônomo
( ) Emprego fixo federal/ estadual/ municipal
( ) Desempregado (a)
( ) Aposentado (a)

7. Aderência a dieta sem glúten

( ) Sempre
( ) Quase sempre
( ) Algumas vezes
( ) Quase nunca
( ) Nunca

8. Renda Mensal

( ) Nenhuma renda.
( ) Menos de 1 salário mínimo

( ) Até 1 salário mínimo
( ) De 1 a 3 salários mínimos
( ) De 3 a 6 salários mínimos
( ) De 6 a 9 salários mínimos
( ) De 9 a 12 salários mínimos
( ) De 12 a 15 salários mínimos
( ) Mais de 15 salários mínimos

9. Você faz uso de antidepressivo?

( ) Sim
( ) Não
Qual ___________

10. Você tem alguma doença grave?

( ) Sim
( ) Não
Qual ___________

Questionário de Qualidade de Vida

Este questionário foi desenvolvido para descobrir como você se sentiu durante as últimas duas semanas. Vamos perguntar a você sobre os sintomas da sua doença celíaca, do seu grau geral de bem-estar e do seu estado emocional. O questionário inclui 28 perguntas. Cada pergunta tem sete respostas possíveis classificadas de 1 a 7. Por favor, leia cuidadosamente cada pergunta e marque a resposta que melhor descrever como você se sentiu durante as duas últimas semanas. Se um item não se aplica a você (como por exemplo, se teve atividade sexual), deixe a pergunta sem resposta.

1. Quantas vezes nas últimas duas semanas a sua vida foi afetada por uma necessidade urgente de ir ao banheiro por causa de uma vontade súbita de evacuar?
    1. Sempre
    2. Quase sempre
    3. Várias vezes
    4. Algumas vezes
    5. Poucas vezes
    6. Quase nunca
    7. Nunca
2. Com qual frequência nas últimas duas semanas você se sentiu fisicamente exausto(a) ou cansado(a).
    1. Sempre
    2. Quase sempre
    3. Várias vezes
    4. Algumas vezes
    5. Poucas vezes
    6. Quase nunca
    7. Nunca
3. Com qual frequência nas últimas duas semanas você se sentiu frustrado(a), impaciente ou irrequieto(a)?
    1. Sempre
    2. Quase sempre
    3. Várias vezes
    4. Algumas vezes
    5. Poucas vezes
    6. Quase nunca
    7. Nunca
4. Quantas vezes nas últimas duas semanas você recusou ou evitou um convite para jantar com família ou amigos devido a sua doença celíaca?
    1. Todas as vezes

2. Quase sempre
3. Boa parte das vezes
4. Algumas vezes
5. Poucas vezes
6. Quase nunca recusei
7. Nenhuma vez

5. Com qual frequência nas últimas duas semanas o seu intestino ficou solto?
    1. Sempre
    2. Quase sempre
    3. Várias vezes
    4. Algumas vezes
    5. Poucas vezes
    6. Quase nunca
    7. Nunca
6. Com que frequência nas últimas duas semanas, você se sentiu disposto(a), ativo(a), com energia física e mental para realizar tarefas ou trabalhar?
    1. Sem disposição alguma
    2. Muita pouca disposição
    3. Pouca disposição
    4. Alguma disposição
    5. Uma boa quantidade de disposição
    6. Muita disposição
    7. Cheio(a) de disposição
7. Quantas vezes durante as últimas duas semanas você se preocupou com a possibilidade de seu(s) filho(os/as) virem a herdar ou terem de herdar a sua doença celíaca?
    1. Sempre
    2. Quase sempre
    3. Várias vezes
    4. Algumas vezes
    5. Poucas vezes
    6. Quase nunca
    7. Nunca
8. Quantas vezes nas últimas duas semanas você sofreu com cólicas abdominais?
    1. Sempre
    2. Quase sempre
    3. Várias vezes
    4. Algumas vezes
    5. Poucas vezes
    6. Quase nunca
    7. Nunca
9. Você teve alguma dificuldade em praticar atividades recreativas ou esportes devido à sua doença durante as últimas duas semanas?
    1. Dificuldade extrema, não pratiquei nenhuma atividade recreativa ou esportiva
    2. Dificuldade mais que considerável em praticar atividade recreativa ou esportiva
    3. Dificuldade considerável em praticar atividade recreativa ou esportiva
    4. Alguma dificuldade em praticar atividade recreativa ou esportiva
    5. Pouca dificuldade em praticar atividade recreativa ou esportiva
    6. Quase nenhuma dificuldade em praticar atividade recreativa ou esportiva

7. Nenhuma dificuldade, a doença celíaca não afetou minhas atividades esportivas ou recreativas.

10. Com qual frequência nas últimas duas semanas você se sentiu deprimido(a) ou desanimado(a).
    1. Sempre
    2. Quase sempre
    3. Várias vezes
    4. Algumas vezes
    5. Poucas vezes
    6. Quase nunca
    7. Nunca

11. Quantas vezes durante as duas últimas semanas você sofreu por excesso de gases ou sentiu sua barriga inchada?
    1. Sempre
    2. Quase sempre
    3. Várias vezes
    4. Algumas vezes
    5. Poucas vezes
    6. Quase nunca
    7. Nunca

12. Pessoas com doença celíaca muitas vezes têm preocupações e receios relacionados à sua doença. Quantas vezes durante as últimas duas semanas você se preocupou ou teve medo de ter câncer em decorrência de sua doença celíaca?
    1. Sempre
    2. Quase sempre
    3. Várias vezes
    4. Algumas vezes
    5. Poucas vezes
    6. Quase nunca
    7. Nunca

13. Quantas vezes nas últimas duas semanas você sentiu que não esvaziou o seu intestino completamente?
    1. Sempre
    2. Quase sempre
    3. Várias vezes
    4. Algumas vezes
    5. Poucas vezes
    6. Quase nunca
    7. Nunca

14. Quantas vezes nas últimas duas semanas você se sentiu relaxado(a) e livre de quaisquer tensões?
    1. Nunca
    2. Quase nunca
    3. Poucas vezes
    4. Algumas vezes
    5. Várias vezes
    6. Quase sempre
    7. Sempre

15. Quantas vezes nas últimas duas semanas você se sentou isolado(a) ou excluído(a) pelos outros por ser celíaco?
    1. Sempre

2. Quase sempre
3. Várias vezes
4. Algumas vezes
5. Poucas vezes
6. Quase nunca
7. Nunca

16. Com qual frequência nas últimas duas semanas você se sentiu choroso(a) ou chateado(a)?
    1. Sempre
    2. Quase sempre
    3. Várias vezes
    4. Algumas vezes
    5. Poucas vezes
    6. Quase nunca
    7. Nunca
17. Com qual frequência nas últimas duas semanas você teve crise de arrotos?
    1. Sempre
    2. Quase sempre
    3. Várias vezes
    4. Algumas vezes
    5. Poucas vezes
    6. Quase nunca
    7. Nunca
18. Até que ponto a doença celíaca restringiu a sua atividade sexual durante as duas últimas semanas?
    1. Nenhuma atividade sexual por causa da doença celíaca
    2. Considerável restrição devido a doença celíaca
    3. Restrição moderada devido a doença celíaca
    4. Alguma restrição devido a doença celíaca
    5. Pouca restrição devido a doença celíaca
    6. Quase nenhuma restrição devido a doença celíaca
    7. Nenhuma restringir devido da doença celíaca
19. Quantas vezes nas últimas duas semanas você sofreu de náuseas ou vomito?
    1. Sempre
    2. Quase sempre
    3. Várias vezes
    4. Algumas vezes
    5. Poucas vezes
    6. Quase nunca
    7. Nunca
20. Quantas vezes nas últimas duas semanas você sentiu que pessoas importantes na sua vida como família e amigos demonstraram falta de entendimento com relação a doença celíaca?
    1. Sempre
    2. Quase sempre
    3. Várias vezes
    4. Algumas vezes
    5. Poucas vezes
    6. Quase nunca
    7. Nunca
21. Com qual frequência você se sentiu feliz, contente ou satisfeito(a) com sua vida pessoal nas últimas duas semanas?
    1. Muito insatisfeito(a), infeliz na maior parte das vezes
    2. Geralmente insatisfeito e infeliz

3. Um pouco insatisfeito e infeliz
4. Geralmente satisfeito e feliz
5. Quase sempre satisfeito e feliz
6. Quase sempre muito satisfeito e feliz
7. Muito satisfeito(a), não poderia estar mais feliz ou mais contente

22. Com qual frequência nas últimas duas semanas você sentiu que seus/suas colegas de trabalho ou superiores demostraram falta de entendimento com relação a sua doença celíaca?
    1. Sempre
    2. Quase sempre
    3. Várias vezes
    4. Algumas vezes
    5. Poucas vezes
    6. Quase nunca
    7. Nunca
23. Quantas vezes nas últimas duas semanas você sentiu-se limitado(a) em sua formação profissional ou em sua carreira por causa de sua doença celíaca.
    1. Sempre
    2. Quase sempre
    3. Várias vezes
    4. Algumas vezes
    5. Poucas vezes
    6. Quase nunca
    7. Nunca
24. Quantas vezes nas últimas duas semanas você sentiu-se sobrecarregado com as despesas ou tempo necessário para obter comida sem glúten?
    1. Sempre
    2. Quase sempre
    3. Várias vezes
    4. Algumas vezes
    5. Poucas vezes
    6. Quase nunca
    7. Nunca
25. Quantas vezes durante as últimas duas semanas você sentiu-se sobrecarregado pelos problemas com sua saúde ou com a eficácia das entidades provedoras de assistência à sua saúde, em relação às despesas para a obtenção de alimentos sem glúten e a outras possíveis terapias para a doença celíaca?
    1. Sempre
    2. Quase sempre
    3. Várias vezes
    4. Algumas vezes
    5. Poucas vezes
    6. Quase nunca
    7. Nunca
26. Quantas vezes durante as duas últimas semanas você percebeu falta de competência de seus médicos em relação à doença celíaca?
    1. Sempre
    2. Quase sempre
    3. Várias vezes
    4. Algumas vezes
    5. Poucas vezes
    6. Quase nunca
    7. Nunca

27. Quantas vezes nas últimas duas semanas você se preocupou com a possibilidade que a sua doença celíaca foi diagnosticada tarde demais?
    1. Sempre
    2. Quase sempre
    3. Várias vezes
    4. Algumas vezes
    5. Poucas vezes
    6. Quase nunca
    7. Nunca
28. Quantas vezes nas últimas duas semanas você ficou com medo de exames médicos relacionados a sua doença celíaca como exame de sangue ou endoscopia?
    1. Sempre
    2. Quase sempre
    3. Várias vezes
    4. Algumas vezes
    5. Poucas vezes
    6. Quase nunca
    7. Nunca

O questionário deve demorar em torno de 10 minutos para preencher. O paciente pode completar o questionário sozinho ou com o auxílio de um profissional da saúde, mas sem pedir ajuda para familiares ou amigos.

Emocional (E).

Item 2	+	3	+	6	+	10	+	14	+	16	+	21	=	E

Social (S).

Item 4	+	9	+	15	+	18	+	20	+	22	+	23	=	S

Preocupações (Wo).

Item 7	+	12	+	24	+	25	+	26	+	27	+	28	=	Wo

Gastrointestinal (GI).

Item 1	+	5	+	8	+	11	+	13	+	17	+	19	=	GI

Total (To).

E	+	S	+	Wo	+	GI	+	To
